# XLF-mediated NHEJ activity in hepatocellular carcinoma therapy resistance

**DOI:** 10.1186/s12885-017-3345-y

**Published:** 2017-05-19

**Authors:** Sitian Yang, Xiao Qi Wang

**Affiliations:** 1 Department of Surgery, The University of Hong Kong, 21 Sassoon Road, Hong Kong, China; 2 State Key Laboratory for Liver Research, The University of Hong Kong, Hong Kong, China

**Keywords:** XLF, NHEJ activity, DNA repair, Chemoresistance, HCC

## Abstract

**Background:**

DNA repair pathways are used by cancer cells to overcome many standard anticancer treatments, causing therapy resistance. Here, we investigated the role of XRCC4-like factor (XLF), a core member of the non-homologous end joining (NHEJ) repair pathway, in chemoresistance in hepatocellular carcinoma (HCC).

**Methods:**

qRT-PCR analysis and western blotting were performed to detect expression levels of genes and proteins related to NHEJ. NHEJ repair capacity was assessed in vitro (cell-free) and in vivo by monitoring the activity of the NHEJ pathway. Cell viability and IC50 assays were used to measure sensitivity to drug therapy. A xenograft HCC model was used to develop methods of targeting XLF-induced chemosensitization. Clinicopathological analysis was conducted on patients with HCC treated with transarterial chemoembolization (TACE).

**Results:**

Many conventional cancer chemotherapeutics induce DNA double-strand breaks (DSBs). HCC cells respond to these breaks by increasing their NHEJ activity, resulting in resistance. XLF-knockdown cells show an inhibition of NHEJ activity in both cell-free and live-cell assays as well as a high level of unrepaired cellular DSBs. These results indicate that XLF facilitates DNA end-joining and therefore promotes NHEJ activity in cancer cells. Consequently, knockdown of XLF significantly chemosensitized resistant cells both in vitro and in xenograft tumors. A low rate of XLF genomic alteration was found in patients with primary HCC, but XLF expression was induced after drug treatment. Clinically, a high level of XLF expression is significantly associated with advanced HCC and shorter overall survival.

**Conclusion:**

Chemotherapy-induced overexpression of XLF and XLF-mediated enhancements in NHEJ activity contribute to chemoresistance in HCC cells and patients with HCC. Targeting XLF to modulate DSB repair could enhance drug sensitivity and may be a therapeutically useful addition to conventional therapy.

**Electronic supplementary material:**

The online version of this article (doi:10.1186/s12885-017-3345-y) contains supplementary material, which is available to authorized users.

## Background

Many chemotherapeutic drugs induce DNA damage. Drug-induced DNA lesions are recognized by DNA damage response (DDR) factors, which activate cell cycle checkpoints and direct DNA repair pathways. These events enable tumor cells to survive chemotherapy. Therefore, the effectiveness of DNA-damaging drugs largely depends on the DNA damage repair capacity of a cancer cell. Using a combination of DNA-damaging drugs and drugs targeting DDR and DNA damage repair pathways is an obvious therapeutic strategy for cancer, not only to enhance therapeutic sensitivity but also for targeted cancer therapy [1–4]. Current DDR-targeting therapies include (a) restoration of wild type p53 activity, as commonly occurring mutations can inactivate p53 and consequently abrogate cell cycle checkpoints; (b) direct inhibition of cell cycle checkpoint regulators, leading to cell death; and (c) inhibition of DNA damage repair pathways, causing general disease-associated deregulation of DNA repair [1, 4].

Cancers often develop defects in genes associated with DNA repair pathways. Therapeutic interventions targeting proteins with functions dispensable for normal cells but essential for cancer cell survival provide synthetic sensitivity or lethality (SSL) [4, 5]. As expected, due to the severe consequences associated with unrepaired DSBs, the homologous recombination (HR) and non-homologous end joining (NHEJ) pathways display a high level of SSL with many other pathways. For example, PARP1, a PARP inhibitor, creates synthetic lethality in HR-deficient cancers, such as breast and ovarian tumors harboring BRCA1 and BRCA2 gene mutations [2, 4, 6]. However, resistance to PARP inhibitors has been reported [7], and crosstalk between DNA repair pathways such as the HR and NHEJ pathways could result in the acquisition of resistance mechanisms in tumors [3]. Thus, the identification of novel DDR targets is needed and requires further investigation [4].

Direct double-strand breaks (DSBs) are considered the most lethal type of DNA lesion [8]. DSBs induced by ionizing radiation (IR) and radiomimetic drugs are mainly repaired by NHEJ [9], whereas replication-associated DSBs are repaired by HR and related replication repair pathways [10]. Unlike HR, which is restricted in the S and G2/M phases of the cell cycle, NHEJ is active throughout the cell cycle and is the only DSB repair mechanism available in G1, during which there is no template for HR [1]. Well-characterized core members of the NHEJ pathway include KU70/KU80, DNA-dependent kinase (DNA-PKcs), Artimes, ligase 4 (LIG4), X-ray-cross-complementation gene 4 (XRCC4), and XRCC4-like factor (XLF). DNA-PKcs regulatory subunits KU70 and KU80 bind to DSB ends and dictate NHEJ pathway choice. In association with XRCC4 and XLF, LIG4 ligates exposed ends of DNA [1, 11]. A recently identified component of the NHEJ pathway in human cells is PAXX (Paralog of XRCC4 and XLF), which functions in concert with XRCC4 and XLF to mediate DSB repair [12]. XLF (also called Cernunnos or NHEJ1) has recently been identified as a core NHEJ factor. XLF-null human cells are highly sensitive to IR and have profound DBS repair defects. The absence of XLF also leads to V(D)J recombination defects (13, 14).

Chemotherapy is a principal treatment for cancer, but resistance to chemotherapy drugs and molecular targeted therapeutics creates a major obstacle for cancer therapy. Hepatocellular carcinoma (HCC) is a highly chemoresistant cancer with limited therapeutic options. Many types of cancers have been shown to possess DDR gene mutations and DDR pathway gene dis-regulation [1, 4]; however, these features have not been identified in HCC [4]. Thus, for HCC, whether the DDR pathway is deregulated and how this pathway is related to therapy resistance are not well defined. In this study, we investigated the roles of XLF-mediated NHEJ in drug response and resistance in HCC.

## Methods

### Cell culture and drug sensitivity

The HCC cell lines PLC/PRF/5 (ATCC, CRL-8024), Huh7 (provided by Dr. H Nakabayashi, Hokkaido University, Japan), MHCC97 L (97 L) and MHCC97H (97H) (provided by Liver Cancer Institute of Fudan University, China) were cultured in DMEM containing 10% FBS. CD133-PE-labeled Huh7 cells were sorted using magnetic microbeads conjugated to an anti-PE antibody (Miltenyi Biotec. Germany). Sorted CD133+ and CD133− cells were cultured for further experiments. The chemotherapeutic drugs cisplatin (cis, Mayne Pharma, Melbourne, Australia), oxaliplatin (oxa, Jiangsu Hengrui Medicine Co., China), doxorubicin (dox, Main Luck Pharmaceuticals, China), and 5-fluorouacial (5FU, Sigma-Aldrich, St. Louis, MO) were applied to cells, and cell viability was determined by incubation with tetrazolium salt (Cell Counting Kit 8, Dojindo, Japan) and using colony-forming assays.

### Transfection

Small interfering RNAs (siRNAs) against human XLF and ERCC1 (Santa Cruz Biotechnology, Dallas, TX) were transfected into 97 L cells using Lipofectamine RNAiMAX (Life Technologies, Carlsbad, CA). Scrambled siRNA was used as a negative control. shRNA against human NHEJ1 and a scrambled control were constructed using a pEco-Lenti-H1-shRNA (GFP) kit (GenTarget Inc., San Diego, CA). Lentivirus particles were produced in 293 T cells using ViraPower Lentiviral Packaging Mix (Life Technologies) and concentrated by ultracentrifugation (20,000 g). 97 L cells were infected with shXLF or shCon lentiviral particles, followed by puromycin selection for 7–10 days.

### Antibodies, western blotting, and immunofluorescence

A phospho-histone H2AX (Ser139) (γH2AX) antibody was obtained from Cell Signaling Technology (Beverly, MA). An antibody against Ligase IV was purchased from Santa Cruz Biotechnology (Santa Cruz Biotechnology, Dallas, Texas), and an ERCC1 antibody was purchased from Thermo Fisher Scientific (Waltham, MA). For western blotting (WB), PVDF membranes containing proteins electrophoretically separated from cell lysates were probed with relevant antibodies. The resultant immune complexes were visualized using enhanced chemiluminescence detection reagents (Bio-Rad, Hercules, CA). For immunofluorescence staining, cells were harvested and cyto-spun, followed by fixation with 4% paraformaldehyde. After blocking and permeabilization with 1%BSA/0.3%Triton-X100 in PBS for 1 h, the cells were incubated with anti-γH2AX antibody overnight at 4 °C. A FITC-conjugated secondary antibody (Life Technologies) was utilized to visualize the signal.

### Cell-free (in vitro) and living cell (in vivo) NHEJ assays

For the in vitro NHEJ assay, cellular nuclear protein fractions were isolated and applied to repair DNA DSBs in vitro as previously described [15]. Briefly, cellular nuclear protein was incubated with linearized plasmid DNA in T4 ligase buffer and 1 mM dNTPs for 2 h at 14 °C. After de-proteinization, the quantity of end-joined DNA products was measured by quantitative PCR with a pair of primers flanked primer-joining junction of the plasmid. Relative NHEJ activity was calculated as the ratio of end-joined products normalized to the loading control. For the in vivo NHEJ assay, the engineered construct pEGFP-PEM1-Ad2 [16] was transfected using X-tremeGENE HP reagent (Roche, Hong Kong). The starting construct was GFP-negative, and the successful repair of Hind III-induced DSBs by cellular NHEJ was identified by restored functionality of the GFP gene [16]. After transfection, the number of GFP-positive cells was measured by flow cytometry and normalized to the transfection efficiency for in vivo NHEJ activity.

### Xenograft tumor model

97 L cells (1 × 10^6^) infected with lentiviruses harboring shRNA-XLF or shRNA-scramble were subcutaneously injected into nude mice to generate an HCC xenograft model. Two weeks after tumor cell injection, 4 cycles of oxaliplatin (4–10 mg/kg) were given via weekly intraperitoneal injection. Tumor volumes were measured at the endpoint. Nuclear proteins from the xenograft tumors were extracted to perform cell-free NHEJ assays. All animal experiments were approved by the Committee on the Use of Live Animals of The University of Hong Kong (CULATR 3091–13).

### HCC tumor specimens

Tissue specimens were collected from 31 patients with HCC who received transarterial chemoembolization (TACE) as a first treatment followed by a hepatectomy. All patients were treated at Queen Mary Hospital. Tissue specimens were also collected from patients with HCC who underwent hepatectomy as a first treatment. This study was approved by the Institutional Review Board of the University of Hong Kong/Hospital Authority of Hong Kong (UW05–3597/I022). The need for informed consent was waived because the study was retrospective in design.

### Statistical analysis

Data are presented as the mean ± SD. Paired and independent Student’s t tests were performed using SPSS 21 software (IBM Corp. Armonk, NY). Overall and disease-free survival rates for the included patients were analyzed using the Kaplan-Meier log-rank method.

## Results

### Therapy sensitivity is associated with NHEJ activity

**Fig. 1:**
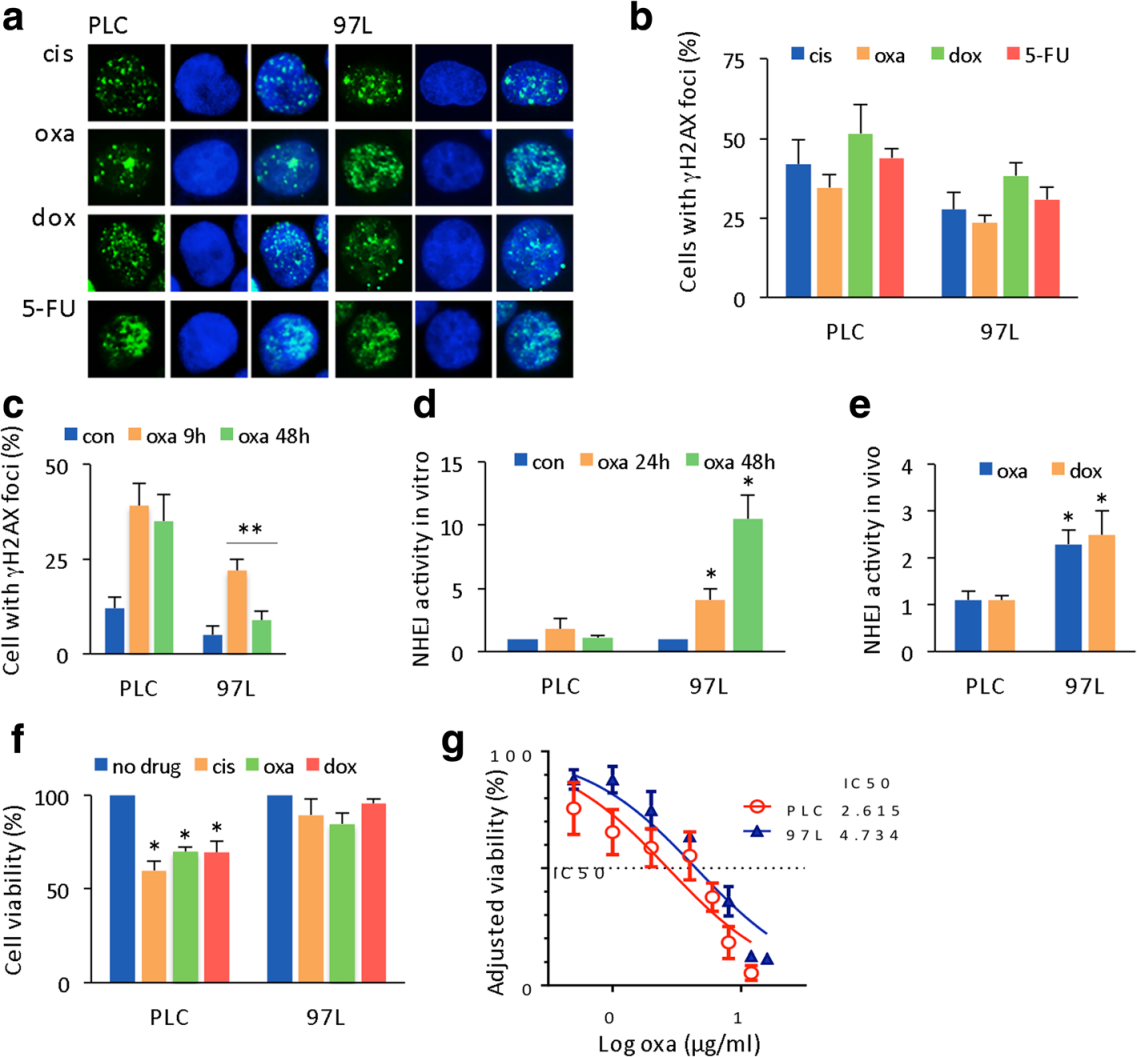
Chemosensitivity in HCC cells is associated with drug-induced increases in NHEJ activity. **a** Following conventional therapy regimens with the drugs cisplatin (cis), oxaliplatin (oxa) doxorubicin (dox), as well as treatment with 5-FU, DSB foci in PLC and 97 L cells were localized using an anti-γH2AX antibody. **b** Quantification of the percentage of γH2AX foci-positive cells. Data are reported as the mean ± SD from 2 independent experiments. **c** Statistical comparison of the percentage of γH2AX-positive cells between PLC and 97 L groups at 9 h and 48 h after drug treatment. A total of 1000 cells were randomly selected for counting. The mean ± SD was from 3 independent experiments. *p* values ≤0.05 and ≤0.01 were denoted as * and **, respectively. **d** In vitro NHEJ activity was assayed after oxaliplatin and doxorubicin treatment and quantified by plasmid-based quantitative PCR (see methods). NHEJ activity from 3 independent experiments was statistically analyzed using a paired Student’s t test. **e** HCC cells were transfected with the plasmid pEGFP-PEM1-Ad2, followed by drug treatment. In vivo NHEJ activity was calculated based on the frequency of GFP-positive cells normalized to the transfection efficiency. Drug-induced NHEJ activity was counted as a ratio of drug-induced activity versus control activity. Representative data are reported as the mean ± SD, *n* = 3. **f** PLC and 97 L cells were treated with cisplatin (1 μg/ml), oxaliplatin (1 μg/ml), or doxorubicin (0.2 μg/ml). Cell viability (%) was determined using a Cell Counting Kit 8 and standardized against cells without drug treatment. Representative data are reported as the mean ± SD, *n* = 3. **g** The therapy drug (oxaliplatin) response curve and the IC50 concentration for the PLC and 97 L cells were analyzed from 2 independent experiments using GraphPad 6.0

To better understand how DNA damage repair contributes to HCC therapy resistance, we first investigated the correlation between the presence of DNA lesions induced by chemotherapeutic drugs and cellular sensitivity. To accomplish this, three types of drugs with different DNA-damaging mechanisms were applied. γH2AX nuclear foci staining was used to detect DSBs: within minutes of the occurrence of DNA damage, H2A.X becomes phosphorylated at Ser139 (γH2AX) at sites of damage [17]. Cisplatin and oxaliplatin (DNA crosslinking agents), doxorubicin (topoisomerase II inhibitor), and 5-FU (antimetabolites) can all induce DSBs both directly and indirectly (Fig. 1a). Following treatments with these drugs, 25% to 50% of 97 L and PLC cells showed positive staining for γH2AX in nuclear foci (Fig. 1b). The γH2AX-positive nuclear foci peaked at 9 h after drug treatment (Fig. 1c). Interestingly, at 48 h, the percentage of γH2AX-positive cells in the 97 L cell group was significantly reduced (Fig. 1c, right panel) compared to that in the PLC cell group, where the percentage remained high (Fig. 1c, left panel). This result indicates that these two cell lines harbor differences in their DSB repair capacity.

We further compared NHEJ activity in vitro and in vivo. Nuclear proteins were isolated from drug-treated cells to perform in vitro NHEJ assays using double-stranded plasmid DNA as a substrate. NHEJ activity in 97 L cells was 5- to 10-fold higher in cells submitted to drug treatment compared to untreated cells. In contrast, NHEJ activity in PLC cells did not change in response to drug treatment (Fig. 1d). Similarly, the drugs oxaliplatin and doxorubicin induced NHEJ activity in 97 L cells in live-cell assays but not in PLC cells (Fig. 1e). Thus, 97 L cells displayed significantly higher in vitro and in vivo NHEJ activity than PLC cells. Concordantly, we found that PLC cells were sensitive to the drugs, whereas 97 L cells were resistant to them (Fig. 1f). The half maximal inhibitory concentration (IC50) for oxaliplatin for inducing HCC cell death was consistently significantly higher in 97 L cells (4.8 μg/ml) than in PLC cells (2.6 μg/ml) (Fig. 1g). Thus, conventional chemotherapeutic drugs for HCC treatment can induce DSBs, and NHEJ responses following treatment with DNA-damaging drugs differ among different cells. HCC cells (97 L) with increased NHEJ activity are more resistant to drugs, whereas HCC cells (PLC) with no enhancement of NHEJ activity are more sensitive.

### Liver CSCs possess high NHEJ capacity

**Fig. 2:**
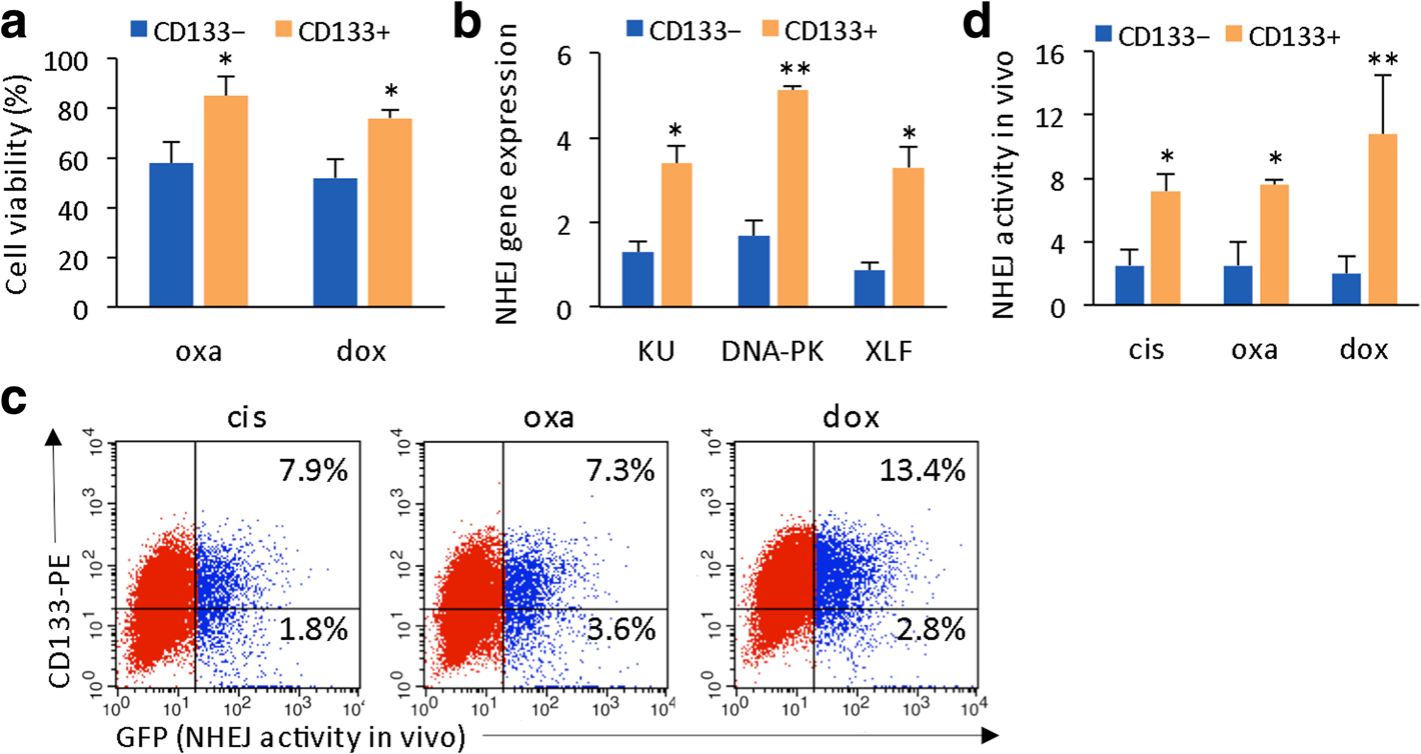
Liver CD133+ CSCs possess high intrinsic NHEJ capacity. **a** Statistical comparison of drug sensitivity in CD133+ and CD133− liver cancer cells measured by cell viability assays. **b** Expression levels of the NHEJ genes Ku, DNA-PK, and XLF in CD133+ and CD133− HCC cells determined by qRT-PCR. Expression levels were normalized to the reference gene 18S rRNA. The data are reported as the mean ± SD obtained from 2 experiments with duplicates. **c** Huh7 cells were transfected with pEGFP-PEM1-Ad2 plasmid, followed by cisplatin, oxaliplatin, or doxorubicin treatment. GPF+ gating was based on CD133-PE-positive (upper part) and CD133-PE-negative (lower part) populations. Live-cell NHEJ activity is presented as the percentage of GFP+ cells within CD133+ and CD133− cell populations and was normalized to the transfection efficiency. **d** Statistical comparison of drug-induced NHEJ activity in vivo of **c** between CD133+ and CD133− cells from 2 independent experiments

Cancer stem cells (CSCs) are highly resistant to chemotherapeutic drugs [18, 19]. We found that a correlation exists between relative chemotherapeutic response and NHEJ activity in HCC CD133+ (potential CSC population) and CD133− populations. In studying Huh7-CD133+ and Huh7-CD133− populations, we found that the former was more drug resistant than the latter, with 75–85% of CD133+ cells surviving after oxaliplatin or doxorubicin treatment (Fig. 2a). When investigating whether NHEJ mechanisms contribute to liver CSC resistance, we found that gene expression levels of the NHEJ factors Ku, DNA-PKcs, and XLF were significantly higher in CD133+ than in CD133− cells (Fig. 2b). We next transfected pEGFP-PEM1-Ad2 plasmids into both CD133+ and CD133− populations and then observed drug-induced NHEJ activity in vivo. The CD133+ cells contained a significantly higher GFP+ population than the CD133− cells (Fig. 2c), and this pattern was consistent with all three drug treatments (Fig. 2c). This result indicates that DSB repair capacity in CD133+ cells is higher than in CD133− cells. Statistical comparisons of the in vivo NHEJ assay results are summarized in Fig. 2d. The results indicate that significantly higher NHEJ activity is found in CD133+ cells. These results suggest that CSCs possess higher intrinsic NHEJ capacity.

### XLF knockdown enhances chemosensitivity in HCC cells

**Fig. 3:**
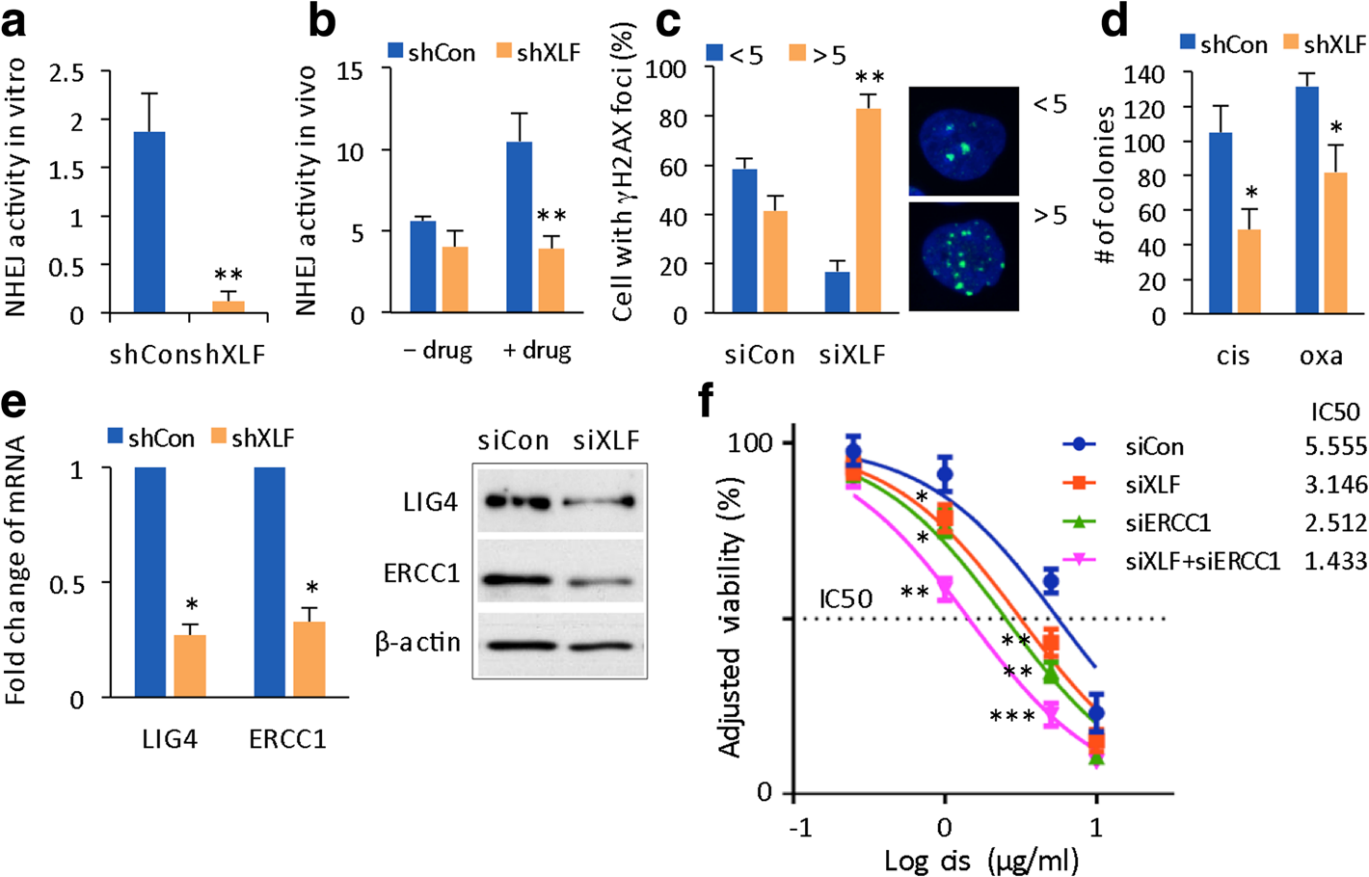
Knockdown of XLF chemosensitizes resistant 97 L cells by causing inhibition of NHEJ activity. **a** In vitro NHEJ activity was assayed and quantified in shRNA-Con and shRNA-XLF lentivirus-infected 97 L cells from 3 independent experiments. **b** In vivo NHEJ activity was calculated as the percentage of GFP-positive cells and normalized to transfection efficiency for the shCon and shXLF group, which were transfected with pEGFP-PEM1-Ad2 plasmid and treated with or without oxaliplatin. The data are reported as the mean ± SD, *n* = 4. **c** Quantification of the percentage of cells with <5 and >5 γΗ2ΑX foci per nucleus at 26 h after drug treatment for the siRNA-Con and siRNA-XLF groups (left panel). The data are reported as the mean ± SD, *n* = 2. Image of γH2AX speckles with <5 and >5 foci per nucleus (right panel). **d** Statistical comparison of drug sensitivity based on colony formation assay results from shCon and shXLF cells. Number of colonies is reported as the mean ± SD from 2 independent experiments. **e** Gene expression levels (left panel) and protein levels (right panel) of LIG4 and ERCC1 were determined by qRT-PCR and WB, respectively, in shCon and shXLF HCC cells. **f** Cisplatin response curves and IC50 concentrations for siCon, siXLF, siERCC1, and siXLF + siERCC1 cells, respectively. Statistical comparison was between siXLF, siERCC1, and siXLF + siERCC1 versus siCon, respectively, with treatment by cisplatin 1 or 5 μg/ml. * *p* < 0.05; ** *p* < 0.01; *** *p* < 0.001

XLF is a core member of the NHEJ protein complex. XLF interacts with XRCC4 to form an XRCC4/XLF dimer that bridges DNA ends in vitro; thus, XLF might stimulate ligation [20–22]. XLF also accumulates at sites with DSBs [14]. Whether the known functions of XLF have a role in drug-induced DSB repair and cancer resistance is not known. Resistant 97 L HCC cells displayed high NHEJ activity both in vitro and in vivo (Fig. 1d, e). After knocking down XLF, the drug-induced in vitro NHEJ activity was significantly inhibited (Fig. 3a). In vivo, NHEJ activity was still induced by the drugs in shRNA-control-transfected cells, whereas it was not in cells transfected with shRNA-XLF (Fig. 3b). These results imply that down-regulating XLF significantly impairs NHEJ capacity. 97 L cells had higher DSB repair efficiency, and γH2AX foci were reduced after longer-term drug treatment (Fig. 1c). Following XLF knockdown, the number of γH2AX foci remained high, with 83.1% ± 6.1 of cells displaying >5 γH2AX foci (Fig. 3c), indicating the presence of unrepaired DSBs. As a result, 97 L cells became sensitized to the chemotherapeutic drugs tested (Fig. 3d).

### XLF knockdown results in down-regulation of ERCC1

We next explored the cross-regulation of XLF with other DNA repair genes, such as LIG4, as XLF stimulates the XRCC4/LIG4 complex [13]. We also investigated excision repair cross-complementing 1 (ERCC1) of the nucleotide excision repair (NER) pathway. Achieving SSL is ideal for producing therapeutic effects, and the HR and NHEJ pathways show high levels of SSL with the NER pathway [4]. Down-regulation of XLF was associated with reduced LIG4 mRNA (Fig. 3e, left panel) and protein expression levels (Fig. 3e, right panel), which is consistent with previous findings that XLF regulates LIG4 in cancer. Interestingly, in XLF-knockdown cells, ERCC1 mRNA and protein expression levels also decreased (Fig. 3e). We then investigated whether a synergistic effect exists between the chemotherapeutic drugs after two DNA repair genes are inhibited. In combination with ligase inhibitor (L189), the drugs inhibited the growth of shXLF cells more than the growth of shCon cells (Additional file 1: Fig. S1). Inhibiting XLF enhanced the chemotherapeutic sensitivity in resistant 97 L HCC cells (Fig. 3d). We then determined a synergistic effect compared to cisplatin alone, particularly when both XLF and ERCC1 were knocked down because ERCC1 positive-tumors predict cisplatin resistance in non-small-cell lung cancer and squamous cell carcinoma [23–25]. The IC50 of cisplatin was 5.56 μg/ml for the siCon group, 3.15 μg/ml for the siXLF group, 2.51 μg/ml for the siERCC1 group, and 1.43 μg/ml for the siXLF + siERCC1 group (Fig. 3f), indicating a chemosensitization effect when there was knockdown of either XLF or ERCC1, where the most enhanced chemosensitivity was seen with knockdown of both XLF and ERCC1. Crosstalk between DNA repair pathways often results in the acquisition of resistance mechanisms in cancer [3]. This result suggests that targeting independent pathways may produce stronger synergy than targeting the same pathway at multiple points [2].

### XLF knockdown enhances therapeutic efficacy in HCC xenograft model

**Fig. 4:**
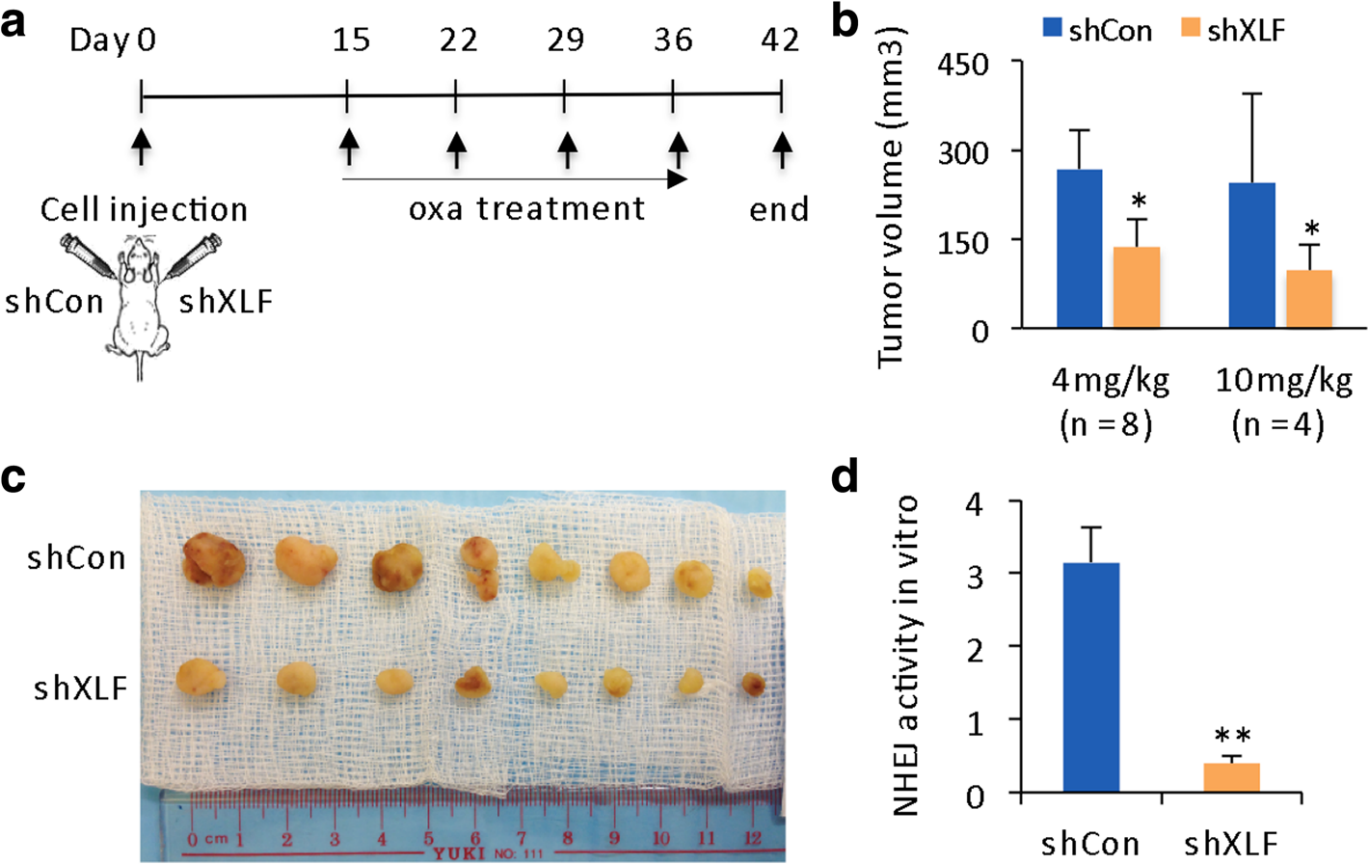
XLF knockdown restores drug sensitivity in HCC xenograft model. **a** Experimental setup used to create the xenograft model. shCon and shXLF 97 L cells (1 × 10^6^) were subcutaneously injected into nude mice. Oxaliplatin (4 mg/kg/week or 10 mg/kg/week) was administered to the mice by intra-peritoneal injection. **b** Tumor volume was reported in mm^3^ as the mean ± SD and statistically compared between shCon and shXLF tumors. **c** Representative images of xenograft tumor. **d** In vitro NHEJ activity in nuclear proteins extracted from xenograft tumor tissue. The statistical comparison was based on the mean ± SD from 3 independent experiments

To establish a xenograft model, shCon- and shXLF-transfected 97 L cells were subcutaneously injected into the left and right flanks, respectively, of the same nude mouse. Using the drug administration protocol depicted in Fig. 4a, oxaliplatin treatment was started at the third week after cell injection and was continued for 4 weeks. At the endpoint, overall tumor volumes were significantly smaller for the shXLF-HCC xenografts than the shCon-HCC xenografts (Fig. 4b, c), indicating that XLF knockdown sensitized HCC xenografts to oxaliplatin. Cell-free NHEJ activity was measured in nuclear proteins extracted from xenograft tissue. A dramatic enhancement in NHEJ activity was observed in the shCon xenografts, whereas NHEJ activity was consistently inhibited in the shXLF xenografts (Fig. 4d). Thus, the shXLF-HCC xenograft tumors were more responsive to oxaliplatin as a result of shXLF-mediated reductions in NHEJ activity.

### Low frequency of XLF gene alterations in patients with HCC

**Fig. 5:**
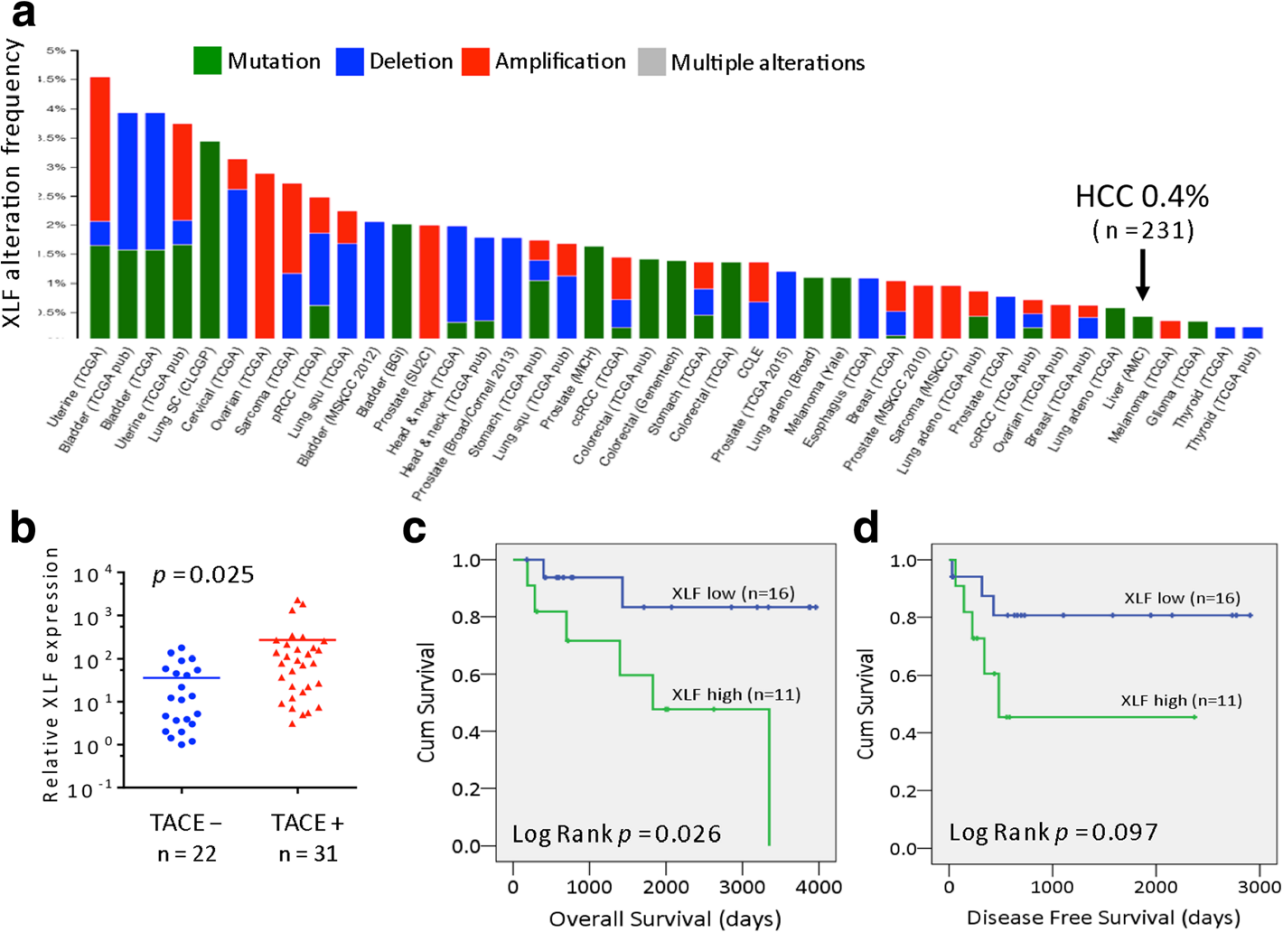
Clinical significance of XLF expression. **a** Genomic alteration frequency for XLF was calculated using cBioPortal [http://www.cbioportal.org] from the TCGA (193 patients) and AMC (231 patients) databases. Tumor types are indicated at the bottom and ordered by the frequency of samples harboring mutation, deletion, amplification and multiple alterations. The XLF mutation rate in HCC is highlighted. **b** Statistical comparison of XLF gene expression in tumor tissue between patients with HCC who underwent TACE (*n* = 31) and those who did not (*n* = 22). **c** Kaplan-Meier analysis of overall survival between patients with HCC with high and low expression of XLF, who all underwent TACE treatment. **d** Kaplan-Meier analysis of disease-free survival for patients with HCC as in **c**

Cancer cells are frequently dysfunctional in one or more DDR and DNA damage repair pathways, which can be specifically inhibited to induce SSL. These dysfunctions can also lead to additional alterations in DDR pathways that may induce therapy resistance [1, 4]. Given that XLF-mediated induction of NHEJ activity in HCC cells is associated with chemotherapeutic drug sensitivity (Figs. 1 and 2), we investigated the incidence of XLF gene alterations using bioinformatic analysis. We also investigated genomic alterations of the core factors in the NHEJ pathway in HCC and other cancers. The NHEJ pathway includes 8 genes: NHEJ1 (XLF), XRCC4, XRCC5, XRCC6, LIG4, DNA-dependent protein kinase catalytic subunit (PRKDC), TP53BP1, and DNA cross-link repair 1C (DCLRE1C). Genomic alterations were identified using cBioPortal [http://www.cbioportal.org] [26, 27] based on 90 studies from 17 to 26 types of primary tumor and cancer lines assembled by The Cancer Genomics Atlas (TCGA) [28] and Asan Medical Center (AMC) [29]. The frequency of alterations, including mutation, deletion, amplification, and multiple alterations, for NHEJ pathway genes in HCC was found to be 17.6% by TCGA and 10% by AMC (Additional file 1: Fig. S2); in contrast, the frequency of alterations in the XLF gene was extremely low in HCC. Indeed, only one of 231 patients had a missense mutation (mutation rate: 0.4%)(Fig. 5a) in the AMC data collection, and no mutations were found in the 371 patients included in the TCGA data collection (Additional file 1: Fig. S3; Additional file 2: Table S1). Moreover, upregulation of XLF mRNA was found in only 5% of patients with HCC (Additional file 2: Table S1). Given the low genomic alteration rate of XLF in patients with HCC, we sought to measure XLF levels in primary HCC tissues from patients who did or did not receive TACE treatment before liver tumor resection to determine whether therapy drugs can impact XLF expression, because TACE delivers chemotherapy in combination with embolization to administer therapy directly to liver tumors. As shown in Fig. 5b, XLF expression was significantly increased in patients with HCC who underwent TACE compared to those who did not, indicating that the delivery of drugs by TACE may directly or indirectly stimulate XLF expression.

### Therapy drugs induce XLF expression and affect clinical outcome

Given that XLF expression (Fig. 5b) and XLF-mediated alterations in NHEJ activity (Fig. 1d, e; Fig. 3a, b) can be induced by therapy drugs, we wanted to assess whether enhancing XLF expression would affect HCC clinical parameters. The patients in the TACE treatment group who showed higher levels of XLF expression displayed poor prognosis features such as larger tumor size, venous infiltration, and advanced-stage HCC disease (Table 1). More importantly, XLF expression was significantly associated with overall survival of HCC patients, with patients exhibiting higher levels of XLF showing shorter survival times (Fig. 5c). High XLF expression was also associated with a shorter disease-free survival time, although the trend did not reach statistical significance (Fig. 5d).

**Table 1:** Correlation between XLF expression and clinicopathologic features in TACE-treated HCC

Clinical features		XLF expression		*P*
		Low	High	
Venous infiltration	(−) (+)	11 2	7 11	0.025*
Size of tumor (cm)	≤5 >5	9 4	5 13	0.022*
No. of nodules	Single Multiple	6 7	8 10	0.925
UICC grade	I, II IIIa, IIIb	12 1	10 8	0.045*
Recurrence	(−) (+)	6 7	6 12	0.47

## Discussion

Cancer cells frequently exhibit dysfunctional DNA repair. Mutations in DNA repair pathways can also be a predisposing factor for cancer. However, once tumors develop, DNA repair pathways can be used by cancer cells to overcome many standard anticancer treatments and are often a cause of chemotherapeutic resistance [30]. DDR pathway deregulation has been analyzed in 15 types of cancer [4], although there have been no previous reports describing this deregulation in HCC [4]. Based on a large dataset assembled by TCGA and AMC with information from 17 to 26 types of primary tumor and cancer lines, we showed here for the first time the genomic alterations that can be found in core factors of NHEJ pathway, including XLF, in patients with HCC. Our analysis demonstrated an extremely low mutation rate (0.4%) for XLF in the AMC data collection and no mutations for XLF in the TCGA data collection for patients with HCC. Furthermore, XLF mRNA overexpression was found in only 5% of patients with HCC. These results suggest that dysregulation of XLF does not contribute to the phenotypic profile of HCC. However, significant upregulation of XLF was observed in patients who underwent TACE, indicating that therapeutic drugs can induce overexpression of XLF. This overexpression was further predictive of poor prognosis and reduced survival. These results led us to develop a new hypothesis: therapy resistance associated with DNA repair can be induced by standard therapeutic treatment and might not be related to pre-existing deregulation of DNA repair pathways. This hypothesis is reinforced by clinical observations that TACE therapy induces robust XLF expression and that XLF induces high levels of NHEJ activity in patients with HCC. These patients might develop DNA repair-mediated therapy resistance, a critical contributor to treatment failure and reduced survival, even after hepatectomy. Thus, rationale for including DNA repair inhibitors as part of the cancer drug armamentarium should be based not only on the presence of abnormally high levels of *intrinsic* expression of DNA repair pathway members in tumor tissue but also on high levels of expression induced by chemotherapeutic drugs.

By comparing two HCC cell lines with different degrees of drug sensitivity, we determined that conventional chemotherapy can induce NHEJ activity in HCC cells. Increases in NHEJ activity leads to the repair of drug-induced DSBs and reduces the presence of cellular γH2AX foci, which significantly decreased the effectiveness of the chemotherapeutic drugs used in this study. Drug-induced activation of NHEJ activity also contributed to the chemoresistance of liver CSCs. We further demonstrated that XLF plays an important role in drug resistance mediated by NHEJ activity. Knocking down XLF significantly enhanced chemosensitization in vitro and in vivo by decreasing NHEJ activity. Thus, for the first time, we have demonstrated that XLF-mediated increases in NHEJ activity are responsible for chemoresistance in HCC cells. The XLF-XRCC4 complex is essential for NHEJ, although how XLF mechanistically functions in NHEJ is not well understood [31]. Recent studies have suggested that XLF-XRCC4 filaments provide both protection to and alignment of DNA ends for accurate and efficient ligation [31, 32]. Whether this mechanism is relevant in our current experimental setting is not known. However, downregulation of XLF significantly impaired ligation and thus reduced NHEJ efficiency, resulting in 80% of γH2AX-positive cells containing >5 γH2AX foci per cell as well as the development of chemosensitization. Moreover, XLF might directly regulate LIG4, as knocking down XLF downregulated LIG4. Together, the roles of the XLF-XRCC4 complex in promoting ligation [31] and XLF-stimulated NHEJ activity in chemoresistance suggests that targeting XLF is a potential avenue for the development of a new DNA repair inhibitor for combination therapy.

DNA-dependent protein kinase catalytic subunit (DNA-PKcs), including Ku70 and Ku80, plays key roles in NHEJ repair for DSB repair and V(D)J recombination. Recent investigations demonstrated that levels of DNA-PKcs were directly associated with genomic stability, cancer cell proliferation index, and patients’ survival length in HCC, suggesting that DNA-PKcs contributes to liver malignant transformation and carcinogenesis. Moreover, elevated DNA-PKcs expression identified HCC patients with therapy-resistance [33, 34]. In combination with our study, intrinsic and drug-induced DNA repair genes of the NHEJ pathway indeed represent prognostic factors and, more importantly, specific therapeutic targets.

γH2AX phosphorylation occurs several minutes after irradiation and DSB formation. Immunological detection of γH2AX indicates the presence of unrepaired DNA breaks. Thus, γH2AX has been utilized as a pharmacodynamic marker in clinical studies. For example, γH2AX foci were enumerated to determine irradiation toxicity at different dosages and to evaluate chemotherapeutic drug responses [4, 35, 36]. According to our observations, the remaining levels but not initial level of γH2AX positive cells is a better predictor for drug sensitivity. Many cancer cells possess high DNA repair capacity, and the number of γH2AX-positive cells and foci after longer-term drug treatment are predictive of cellular response or resistance.

## Conclusion

Overexpression of XLF and increased NHEJ activity mediated by XLF in response to treatment with chemotherapeutic drugs contribute to chemoresistance in HCC cells and patients with HCC. Inhibition of XLF-mediated NHEJ activity results in chemosensitization in a HCC xenograft model, suggesting that XLF is a novel candidate for the development of new DNA repair inhibitors for combination therapy.

## Additional files

Additional file 1: **Figure S1.** Colony formation in shCon or shXLF cells treated with DMSO, cisplatin, cisplatin + L189 (LIG4 inhibitor), oxaliplatin, or oxaliplatin + L189, respectively. **Figure S2.** Genomic alteration frequency of core factors of NHEJ pathway (XLF, XRCC4, XRCC5, XRCC6, LIG4, PRKDC, TP53BP1, and DCLRE1C) was generated using cBioPortal [http://www.cbioportal.org] from database of TCGA (193 patients) and AMC (231 patients). Tumor types are indicated at the bottom and ordered by the frequency of samples harboring mutation, deletion, amplification and multiple alterations. Overall alteration frequency in liver cancer was highlighted. **Figure S3.** RNA-sequencing data organization of NHEJ pathway genes (XLF, XRCC4, XRCC5, XRCC6, LIG4, PRKDC, TP53BP1, and DCLRE1C) by TCGA to show frequency of NHEJ pathway gene amplification, gene expression, missense or truncating mutations. (ZIP 1521 kb)

Additional file 2: Table S1. Alteration of NHEJ pathway genes in HCC by RNA-seq. (DOCX 56 kb)

## Abbreviations

DDR: DNA damage response
DSBs: double strand breaks
ERCC1: excision repair cross-complementing 1
HR: homologous recombination
IC50: inhibitory concentration
NER: nucleotide excision repair
NHEJ: non-homologous end joining
SSL: synthetic sensitivity or lethality
TACE: transarterial chemoembolization
XLF: XRCC4-like factor
