# Supplementary figures and images for: XLF-mediated NHEJ activity in hepatocellular carcinoma therapy resistance
Source: BMC Cancer. 2017 May 19;17:344. doi: 10.1186/s12885-017-3345-y (PMC5437682; doi:10.1186/s12885-017-3345-y)

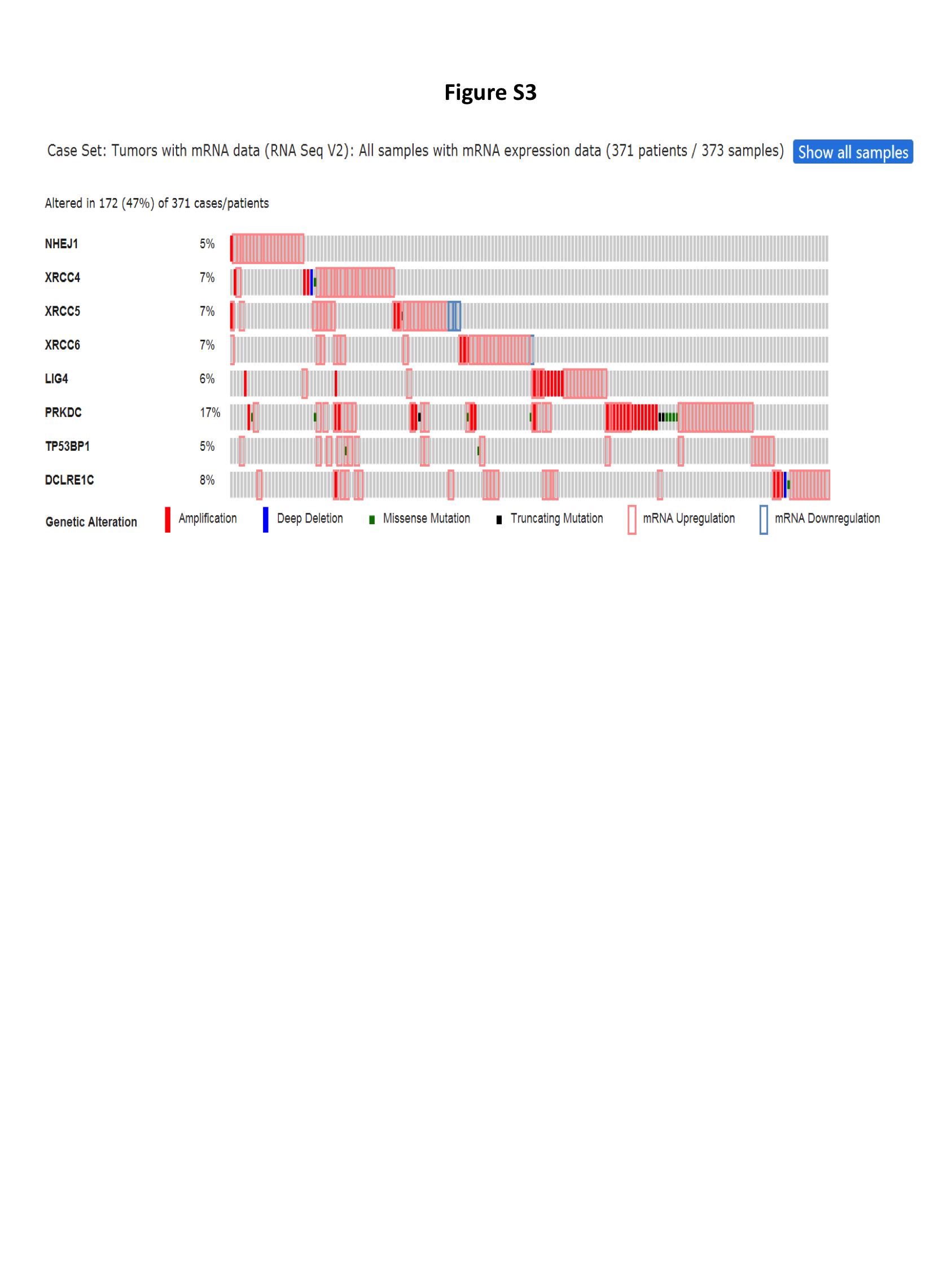

Supplement: Supplementary file 1 — Figure S1. Colony formation in shCon or shXLF cells treated with DMSO, cisplatin, cisplatin + L189 (LIG4 inhibitor), oxaliplatin, or oxaliplatin + L189, respectively. Figure S2. Genomic alteration frequency of core factors of NHEJ pathway (XLF, XRCC4, XRCC5, XRCC6, LIG4, PRKDC, TP53BP1, and DCLRE1C) was generated using cBioPortal [http://www.cbioportal.org] from database of TCGA (193 patients) and AMC (231 patients). Tumor types are indicated at the bottom and ordered by the frequency of samples harboring mutation, deletion, amplification and multiple alterations. Overall alteration frequency in liver cancer was highlighted. Figure S3. RNA-sequencing data organization of NHEJ pathway genes (XLF, XRCC4, XRCC5, XRCC6, LIG4, PRKDC, TP53BP1, and DCLRE1C) by TCGA to show frequency of NHEJ pathway gene amplification, gene expression, missense or truncating mutations. (ZIP 1521 kb) [file 12885_2017_3345_MOESM1_ESM.zip › Figure-S3R6.tiff]

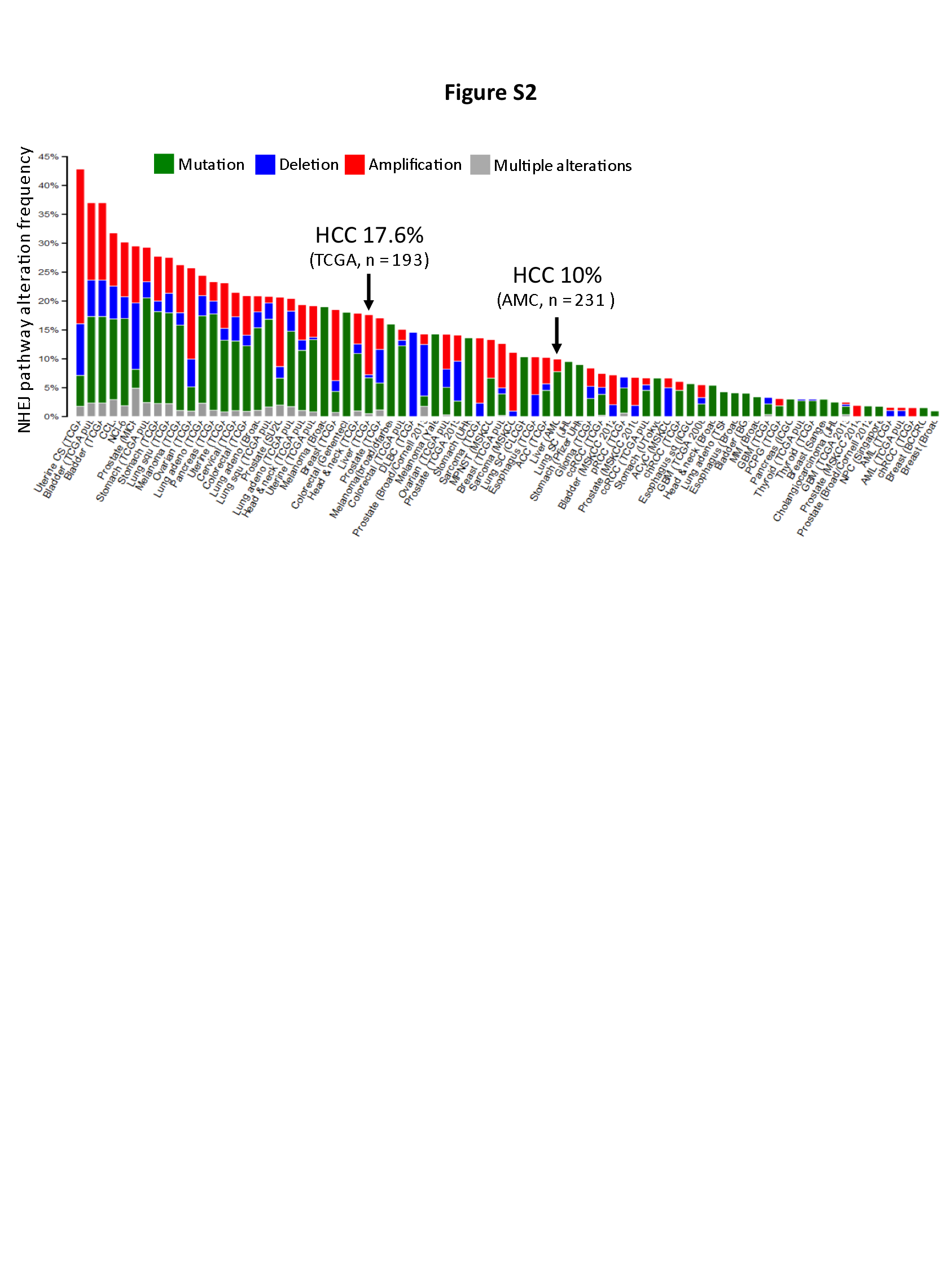

Supplement: Supplementary file 1 — Figure S1. Colony formation in shCon or shXLF cells treated with DMSO, cisplatin, cisplatin + L189 (LIG4 inhibitor), oxaliplatin, or oxaliplatin + L189, respectively. Figure S2. Genomic alteration frequency of core factors of NHEJ pathway (XLF, XRCC4, XRCC5, XRCC6, LIG4, PRKDC, TP53BP1, and DCLRE1C) was generated using cBioPortal [http://www.cbioportal.org] from database of TCGA (193 patients) and AMC (231 patients). Tumor types are indicated at the bottom and ordered by the frequency of samples harboring mutation, deletion, amplification and multiple alterations. Overall alteration frequency in liver cancer was highlighted. Figure S3. RNA-sequencing data organization of NHEJ pathway genes (XLF, XRCC4, XRCC5, XRCC6, LIG4, PRKDC, TP53BP1, and DCLRE1C) by TCGA to show frequency of NHEJ pathway gene amplification, gene expression, missense or truncating mutations. (ZIP 1521 kb) [file 12885_2017_3345_MOESM1_ESM.zip › Figure-S2R6.tiff]

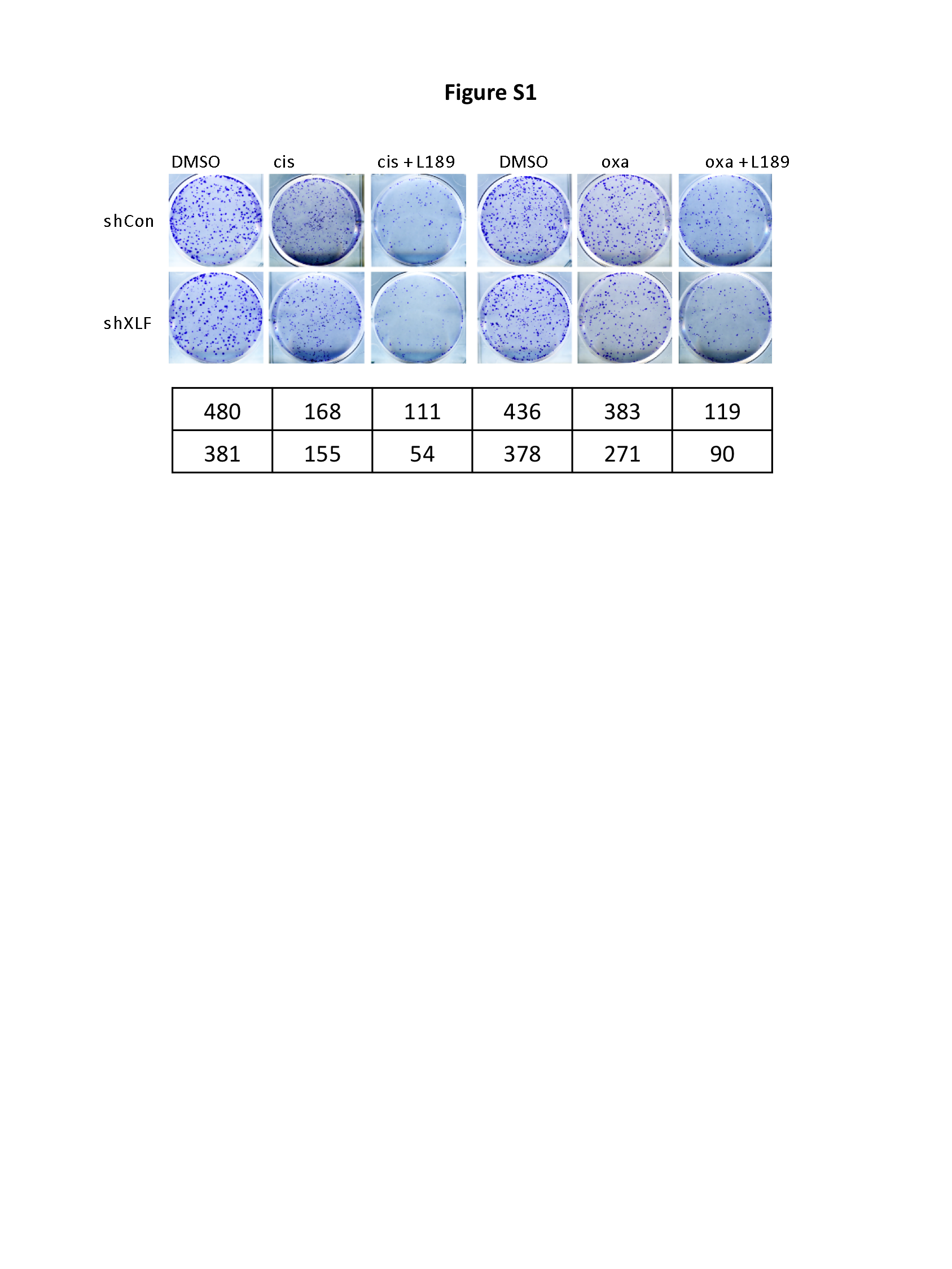

Supplement: Supplementary file 1 — Figure S1. Colony formation in shCon or shXLF cells treated with DMSO, cisplatin, cisplatin + L189 (LIG4 inhibitor), oxaliplatin, or oxaliplatin + L189, respectively. Figure S2. Genomic alteration frequency of core factors of NHEJ pathway (XLF, XRCC4, XRCC5, XRCC6, LIG4, PRKDC, TP53BP1, and DCLRE1C) was generated using cBioPortal [http://www.cbioportal.org] from database of TCGA (193 patients) and AMC (231 patients). Tumor types are indicated at the bottom and ordered by the frequency of samples harboring mutation, deletion, amplification and multiple alterations. Overall alteration frequency in liver cancer was highlighted. Figure S3. RNA-sequencing data organization of NHEJ pathway genes (XLF, XRCC4, XRCC5, XRCC6, LIG4, PRKDC, TP53BP1, and DCLRE1C) by TCGA to show frequency of NHEJ pathway gene amplification, gene expression, missense or truncating mutations. (ZIP 1521 kb) [file 12885_2017_3345_MOESM1_ESM.zip › Figure-S1R6.tiff]
