# Supplementary material for: XLF-mediated NHEJ activity in hepatocellular carcinoma therapy resistance
Source: BMC Cancer. 2017 May 19;17:344. doi: 10.1186/s12885-017-3345-y (PMC5437682; doi:10.1186/s12885-017-3345-y)
Supplement: Supplementary file 2 — Alteration of NHEJ pathway genes in HCC by RNA-seq. (DOCX 56 kb) [file 12885_2017_3345_MOESM2_ESM.docx]

| tcga-hcc | Amplification | Deep deletion | Missense mutation | Truncating mutation | mRNA upregulation | mRNA downregulation | Alteration (%) |
| --- | --- | --- | --- | --- | --- | --- | --- |
| XLF | 1 | 0 | 0 | 0 | 20 | 0 | 5.36 |
| XRCC4 | 3 | 1 | 1 | 0 | 23 | 0 | 7.51 |
| XRCC5 | 3 | 0 | 0 | 1 | 23 | 3 | 7.24 |
| XRCC6 | 3 | 0 | 0 | 0 | 26 | 1 | 7.51 |
| LIG4 | 11 | 0 | 0 | 0 | 17 | 0 | 6.70 |
| PRKDC | 23 | 0 | 8 | 3 | 45 | 0 | 17.69 |
| TP53BP1 | 0 | 0 | 2 | 0 | 18 | 0 | 5.09 |
| DCLRE1C | 4 | 1 | 1 | 0 | 29 | 0 | 8.58 |

Table S1. Alteration of NHEJ pathway genes in HCC (n = 373*) by RNA-seq

* Collection data of TCGA based on HCC 371 patients/373 samples.
